# Supplementary material for: The Next-Generation Probiotic E. coli 1917-pSK18a-MT Ameliorates Cadmium-Induced Liver Injury by Surface Display of Metallothionein and Modulation of Gut Microbiota
Source: Nutrients. 2024 May 13;16(10):1468. doi: 10.3390/nu16101468 (PMC11124084; doi:10.3390/nu16101468)
Supplement: Supplementary file 1 [file nutrients-16-01468-s001.zip › supplementary materials/Table S1 Primer Sequences.docx]

| **cytokines** | **forward prime Sequences** | **reverse prime Sequences** |
| --- | --- | --- |
| GAPDH | 5´-CTCGTGGAGTCTACTGGTGT-3´ | 5´-GTCATCATACTTGGCAGGTT-3´ |
| IL-1β | 5´-GTGTCTTTCCCGTGGACCTTC-3´ | 5´-TCATCTCGGAGCCTGTAGTGC-3´ |
| IL-6 | 5´-CTTCTTGGGACTGATGCTGGTGAC-3´ | 5´-AGGTCTGTTGGGAGTGGTATCCT-3´ |
| TNF-α | 5´-GTGGAACTGGCAGAAGAGGCA-3´ | 5´-AGAGGGAGGCCATTTGGGAAC-3´ |

**Table S1.** **Primer Sequences**
