# Supplementary material for: The Next-Generation Probiotic E. coli 1917-pSK18a-MT Ameliorates Cadmium-Induced Liver Injury by Surface Display of Metallothionein and Modulation of Gut Microbiota
Source: Nutrients. 2024 May 13;16(10):1468. doi: 10.3390/nu16101468 (PMC11124084; doi:10.3390/nu16101468)
Supplement: Supplementary file 1 [file nutrients-16-01468-s001.zip › supplementary materials/Table S2 Antibodies used for Western blotting.docx]

**Table S2.** **Antibodies used for Western blotting**

| **Antibodies** | **Provider** | **Catalogue** | **Host** | **Dilution** |
| --- | --- | --- | --- | --- |
| NF-ĸB p65 | Proteintech | 10745-1-AP | Rabbit | 1:2,000 |
| Phospho-NF-ĸB p65  (p-p65) | Affinity | AF2006 | Rabbit | 1:1,000 |
| Keap1 | Proteintech | 60027-1-lg | Mouse | 1:2,000 |
| Nrf2 | CST | 12721S | Rabbit | 1:1,000 |
| Occludin | Proteintech | 13409-1-AP | Rabbit | 1:1,000 |
| ZO-1 | Proteintech | 21773-1-AP | Rabbit | 1:500 |
| β-actin | CST | 4970S | Mouse | 1:1,000 |
| α-SMA | Proteintech | 14395-1-AP | Rabbit | 1:5,000 |
| GAPDH | Affinity | AF7021 | Rabbit | 1:1,0000 |
